# Wearable Sensors-Based Intelligent Sensing and Application of Animal Behaviors: A Comprehensive Review

**DOI:** 10.3390/s25144515

**Published:** 2025-07-21

**Authors:** Luyu Ding, Chongxian Zhang, Yuxiao Yue, Chunxia Yao, Zhuo Li, Yating Hu, Baozhu Yang, Weihong Ma, Ligen Yu, Ronghua Gao, Qifeng Li

**Affiliations:** 1 Information Technology Research Center, Beijing Academy of Agriculture and Forestry Sciences, Beijing 100097, China; dingly@nercita.org.cn (L.D.); zcx20010327@outlook.com (C.Z.); yueyx_yyx@163.com (Y.Y.); yaocx@nercita.org.cn (C.Y.); lizhuo@nercita.org.cn (Z.L.); huyating@jlau.edu.cn (Y.H.); yangbz@nercita.org.cn (B.Y.); mawh@nercita.org.cn (W.M.); yulg@nercita.org.cn (L.Y.); gaorh@nercita.org.cn (R.G.); 2 National Engineering Research Center for Information Technology in Agriculture (NERCITA), Beijing 100097, China; 3 National Innovation Center of Digital Technology in Animal Husbandry, Beijing 100097, China; 4 College of Engineering, Huazhong Agricultural University, Wuhan 430070, China; 5 College of Information Technology, Jilin Agricultural University, Changchun 130118, China

**Keywords:** behavior monitoring, contact sensing, algorithms, tiny machine learning, monitoring applications

## Abstract

Accurate monitoring of animal behaviors enables improved management in precision livestock farming (PLF), supporting critical applications including health assessment, estrus detection, parturition monitoring, and feed intake estimation. Although both contact and non-contact sensing modalities are utilized, wearable devices with embedded sensors (e.g., accelerometers, pressure sensors) offer unique advantages through continuous data streams that enhance behavioral traceability. Focusing specifically on contact sensing techniques, this review examines sensor characteristics and data acquisition challenges, methodologies for processing behavioral data and implementing identification algorithms, industrial applications enabled by recognition outcomes, and prevailing challenges with emerging research opportunities. Current behavior classification relies predominantly on traditional machine learning or deep learning approaches with high-frequency data acquisition. The fundamental limitation restricting advancement in this field is the difficulty in maintaining high-fidelity recognition performance at reduced acquisition rates, particularly for integrated multi-behavior identification. Considering that the computational demands and limited adaptability to complex field environments remain significant constraints, Tiny Machine Learning (Tiny ML) could present opportunities to guide future research toward practical, scalable behavioral monitoring solutions. In addition, algorithm development for functional applications post behavior recognition may represent a critical future research direction.

## 1. Introduction

As the demand for meat consumption and protein-rich foods rises, annual meat production has increased from 218 million tons during 1997–1999 and is projected to reach 376 million tons by 2030 [1]. This robust demand has driven the rapid advancement of large-scale and intensive livestock production, promoting the development of intelligent and precision livestock farming (PLF), which relies on the perception and analysis of physiological and behavioral information. Monitoring and analyzing of livestock behavior provide the foundation for production evaluation and assist farmers with daily management. For instance, behavior monitoring aids in calculating the feed intake for conducting nutritional assessments of livestock [2,3,4], identifying estrus and parturition in animals [5,6], analyzing health conditions, and providing early warnings for diseases [7,8,9]. Consequently, behavior monitoring has become a crucial aspect of precision livestock farming and the protection of animal welfare.

Traditionally, monitoring animal behavior has relied on the tracking and observation conducted by researchers using stopwatches, telescopes, counters, and other equipment [10]. However, advancements in technology have made behavior monitoring a prominent research area in recent decades, gradually shifting towards more automated and unattended methods through the use of sensors. This paper analyzes the trends in livestock behavior monitoring of livestock and reviewed the state-of-the-art sensing methods and their applications of behavior monitoring using wearable sensors. Based on the overall analysis of the existing research on livestock behavior monitoring using wearable sensors, this paper systematically reviewed: (1) the characteristics and comparisons of different sensing methods for behavior monitoring; (2) the development of algorithms in behavior classification; (3) the exploration of further application after behavior recognition; and finally, (4) summarizes the challenges and future prospects in behavior monitoring of livestock.

## 2. Hotspots in Behavior Monitoring and Application Research

**Figure 1:**
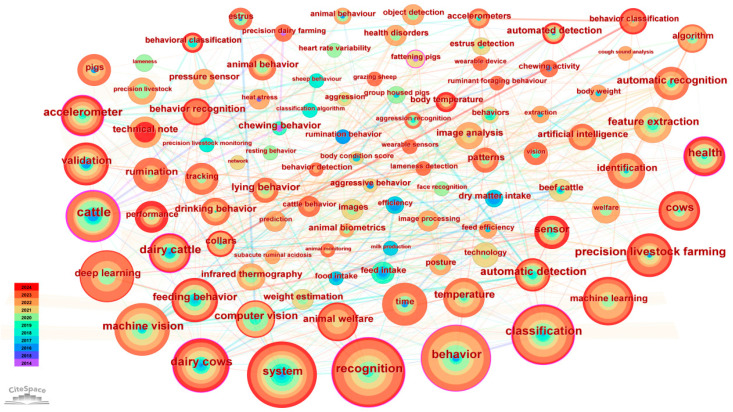
Visualization map of keyword co-occurrence network.

To address the research focal points on livestock behavior monitoring and its applications, this study examines the relevant literature in the Web of Science database from 2014 to 2024 on 30 April 2024. Keywords encapsulate the essence of a paper, providing a high-level summary of its content [11]. The visualization of these keywords facilitates the identification of popular topics and emerging trends within the field [12]. A higher frequency keyword indicates more active research in that area. By utilizing the visualization tool CiteSpace, a knowledge graph of keywords is created to illustrate the hotspots in the latest global research on livestock behavior monitoring. The keywords used in the literature search primarily included “behavior” along with “cattle”, “sheep”, “pigs”, “machine vision”, “sensors”, “feed intake”, and “pasture evaluation”, among others, yielding a total of 185 papers. Figure 1 shows the most frequently appearing keywords in the literature relating to livestock behavior monitoring over the past decade. These keywords can be categorized into three major directions according to their targeting animal species and behavior type, monitoring techniques, monitoring purpose, and application.

### 2.1. Targeting Animal Species and Behavior Type

The targeted monitored animal species include grazing sheep/goats, grazing or housed cattle, and housed pigs, with a primary focus on observing both normal and abnormal behaviors. Table 1 presents the types of target behaviors and their definitions as frequently cited in the literature. The behaviors listed from ‘Feeding’ to ‘Licking/Grooming’ are classified as normal behaviors in Table 1, while ‘Pawing/Kicking’ or ‘Fighting’ are categorized as abnormal behaviors. Specifically, the most studied normal behaviors include feeding, ruminating, resting, walking, and grazing. Most applications of the related technologies are based on monitoring devices attached to the animals’ necks, legs, or ears.

**Table 1:** Definitions of Livestock Behaviors.

Behavior	Definition	Reference
Feeding	Behaviors exhibited by animals while eating, such as chewing and swallowing.	[13,14,15]
Resting	The relaxed state of an animal, lying down, or remaining still, typically to recover energy.	[13,16]
Walking	The behavior of an animal when moving, usually in search of food, water, or other resources.	[13,15,17,18,19,20,21,22]
Standing	The animal maintaining a standing posture, which may be for observing the surroundings, waiting, or preparing to move.	[15,17,18,19,20,21,22,23]
Ruminating	The process in ruminant animals (cattle, sheep) of regurgitating and re-chewing food from the stomach.	[13,15,16,18]
Grazing	Eating forage at ground level with the head down.	[13,14,16,17,19,22]
Socializing	Interactions between animals, such as sniffing or physical contact, typically seen as social behavior in group-living species.	[23,24,25]
Exploring	The behavior of animals investigating their surroundings by sniffing, licking, or observing, especially in new environments or when encountering novel stimuli.	[23,24]
Parturition	Involving uterine contractions, the expulsion of offspring, and observable behaviors such as restlessness, vocalization, and seeking isolation.	[25]
Licking /Grooming	The behavior of licking either their own body or another animal, usually for cleaning or showing affection.	[23]
Pawing /Kicking	The behavior of animals pawing the ground or kicking, often due to agitation or aggressive emotions (rare in pigs).	[24]
Fighting	Intense confrontational behavior between animals, often over resources or status, such as wrestling or headbutting	[24]

### 2.2. Monitoring Techniques

Generally, behavior monitoring can be categorized into two types of techniques: non-contact techniques (e.g., machine vision or computer vision) and contact techniques (e.g., wearable sensors such as accelerometers). These methods are at the forefront of current research.

The non-contact monitoring techniques are primarily based on machine vision and have made significant advancements in automation and intelligence, making them particularly suitable for use in housed environments [26,27]. In contrast to free-range or grazing systems, intensive housing systems confine animals to a relatively small and controlled living space, which facilitates continuous observation and enables the monitoring of multiple animals from a single camera view [28,29,30]. However, several challenges remain to be addressed in non-contact monitoring techniques, particularly in complex scenarios involving variable lighting, occlusion, overlapping, and the continuous tracking of animals across different fields of view. Since this paper focuses on contact techniques, a detailed overview of research developments in non-contact monitoring is not included here.

On the other hand, contact-based monitoring techniques (wearable sensor monitoring) provide a direct and continuous method for recording animal behavior and physiological status. These technologies provide high-resolution and high-accuracy data, which aid in a deeper understanding of animal activity patterns and health conditions. For instance, accelerometers can capture movement patterns, sound sensors can analyze vocalizations to identify changes in emotional or physiological states, and location sensors can track movement paths and area usage [17,31].

Either for non-contact or contact-based monitoring techniques, it relies on subsequent algorithms or models for data processing and analyzing to realize behavior recognition and further application. This is generally associated with techniques in statistical analysis, data mining, and artificial intelligence, including machine learning and deep learning. The effective use of these technologies not only improves the accuracy and efficiency of animal behavior monitoring but also provides essential tools for a deeper understanding of behavior patterns, enhancing animal welfare and farm management.

### 2.3. Monitoring Purposes and Application

**Figure 2:**
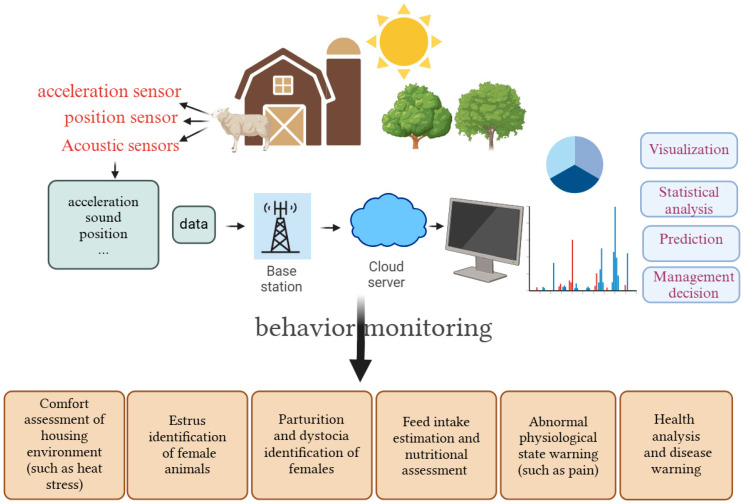
Applications of behavior monitoring for livestock.

From Figure 1, the primary purposes of monitoring mainly include “estrus detection”, “tracking”, “feed efficiency”, and “lameness detection”, etc. For example, assessing the feeding patterns and feed efficiency is crucial for livestock management and animal welfare. By understanding the dietary needs and habits of livestock, farmers can ensure that livestock receive proper nutrition to maintain health and productivity. Lameness is a clinical symptom associated with many sheep diseases worldwide, which not only adversely affects weight gain, fertility, and the birth weight of lambs but also increases the risk of secondary diseases. Barwick et al. [32] used triaxial accelerometers to discriminate lame gait from normal movements in sheep, achieving a maximum recognition accuracy of 87% when the sensor was deployed on the legs. Timely and accurate estrus identification is essential for dairy farm management. Wang et al. [33] developed a dual-channel sound detection tag composed of a monaural microphone and an omnidirectional microphone (OM) for estrus identification of cows. By employing a 70-millisecond time window, Mel-frequency cepstral coefficients (MFCC) features, and a BP neural network algorithm, they achieved an accuracy of 97.62% for estrus identification, demonstrating the potential use of acoustic detection as an alternative way for estrus identification. These monitoring purposes enable intelligent application for early warning and analysis in livestock production, providing base data for smart decision-making [34]. Figure 2 illustrates the various uses of livestock behavior monitoring mentioned in the literature.

## 3. Behavior Monitoring Based on Wearable Sensors

### 3.1. Sensing

#### 3.1.1. Sensor Type

Wearable sensors commonly used for monitoring livestock behavior include inertial sensors (IMU, e.g., triaxial accelerometers, gyroscope sensors), pressure sensors, acoustic sensors, as well as positioning sensors such as BeiDou Navigation Satellite System (BDS), Global Positioning System (GPS), or Global Navigation Satellite System (GNSS). This document provides a review of representative studies on sensor technologies, summarizing the key parameters of these sensors in Table 2. Single-sensor approaches, primarily utilizing accelerometers (ACC), as shown in Table 2, account for the majority of entries. These sensors typically operate at relatively high sampling frequencies (12–62.5 Hz) and are deployed on locations like the neck, ear, or jaw, achieving recognition accuracies normally exceeding 90% for fundamental behaviors such as grazing, walking, resting, and ruminating in both sheep and cattle. However, accuracy can vary significantly depending on the behavior complexity, deployment location, and species. For instance, neck-mounted ACC on cattle showed lower accuracy (80.9–90%) compared to ear-mounted ACC on sheep (94–99%). Acoustic sensors (ACSs), sampled at much higher frequencies (44.1 kHz), target detailed oral behaviors like bites and chews but exhibit wider accuracy ranges (76.5% to 99.5%) and often require larger time windows. Pressure sensors (PRSs), frequently placed on the noseband, demonstrate very high accuracy (93–98%) for jaw activities like eating, chewing, and ruminating. Location-based sensors (GNSS/GPSs) focus on broader applications of grazing animals like feeding patterns analysis or estrus recognition but lack reported accuracy metrics. A key limitation of single-sensor systems is their struggle with complex or composite behaviors, reflected in lower accuracy figures for such categories.

In contrast, Table 3 highlights the growing adoption of multi-sensor fusion, combining modalities like accelerometers (ACCs), gyroscopes (GYRs), GPS, pressure sensors (PRSs), and inertial measurement units (IMUs). The most common combination is ACC + GYR, enhancing the recognition of posture-related behaviors (e.g., lying down, standing, walking). Fusion strategies often pair motion sensors (ACC, GYR, IMU) with location (GPS) or pressure (PRS) data, enabling the identification of more complex and nuanced behaviors, including running, gait patterns, or combinations like “chew-bite.” Multi-sensor systems generally achieve higher reported accuracy levels, with most exceeding 87.8% and the top system (IMU + GPS) reaching 98.9–99.9%. Crucially, they operate with significantly smaller time windows, down to 0.5 s, enabling near real-time behavior detection compared to the often longer windows (5 s to 300 s) seen in single-sensor approaches. While offering superior performance in accuracy, multi-sensor systems inherently increase deployment complexity, requiring sensors on multiple body locations (e.g., ears, neck, legs, chin) and managing synchronized data streams, which currently limits their reported scale compared to some large single-sensor studies. Table 2 and Table 3 offer a particularly valuable perspective compared to many review summaries by explicitly structuring the analysis around the fundamental dichotomy of single-sensor approaches versus multi-sensor fusion [35,36]. This clear comparative lens allows for a direct and systematic evaluation of the trade-offs between these two dominant paradigms in wearable animal monitoring. This depth of information provides researchers and practitioners with a much richer understanding of the real-world implementation specifics, performance characteristics, and limitations associated with both single-sensor and multi-sensor strategies documented in the current literature.

**Table 2:** Key Parameters of Different Sensors in Behavior Recognition (Single-Sensor).

Sensing Method	Sampling Frequency	Deployment Location	Behavior Categories Identified	Time Window Size	Recognition Accuracy	Number of Animals	Species	Reference
ACC	62.5 Hz	Jaw	Grazing, rumination, resting	5 s	93%	3	Sheep	[16]
ACC	12 Hz	Ear	Grazing, standing, walking	10 s	94–99%	10	Sheep	[17]
ACC	50 Hz	Neck	Grazing, walking, rumination, resting, drinking	5.12 s	90%	10	Cattle	[13]
ACC	50–62.5 Hz	Neck, Ear	Feeding, walking, resting, ruminating	4.1–5.12 s	80.9–87.4%	27	Cattle	[13]
ACSs	44.1 kHz	Forehead	Grazing, ruminating	300 points	76.5–83.3%	5	Cattle	[37]
ACSs	44.1 kHz	Neck	Mouth open, mouth closed, mixed mouth movements	256 points	99.5%	10	Cattle	[38]
ACSs	\	Forehead	Bites, exclusive chews, chew-bite combinations, exclusive sorting	2048 points	89.62–95.9%	10	Cattle	[39]
PRSs	2 Hz	Reticulorumen	Ruminating, eating, drinking, sleeping	120 s	98%	4	Cattle	[40]
PRSs	10 Hz	Noseband	Ruminating, eating, drinking, other	10 s	93%	60	Cattle	[41]
PRSs	50 Hz	Noseband	Ruminating, eating, other	10 s	96.6%	3	Cattle	[42]
Location (GNSS)	\	Neck	Movement speeds during estrus and non-estrus	\	\	48	Sheep	[43]
Location (GPS)	\	Neck	Tracking to analyze feeding patterns and pasture grazing behavior	\	\	357	Sheep	[14]

ACC = accelerometer, ACSs = acoustic sensors, PRSs = pressure sensors, GPS = global positioning system, GNSS = global navigation satellite system.

**Table 3:** Key Parameters of Different Sensors in Behavior Recognition (Multi-sensor fusion).

Sensing Method	Sampling Frequency	Deployment Location	Behavior Categories Identified	Time Window Size	Recognition Accuracy	Number of Animals	Species	Reference
ACC, UR	50 Hz	Neck	Grazing, eating, walking, running, standing	0.02 s	95%	1	Sheep	[19]
ACC, GPS	60 Hz (GPS), 12 Hz (ACC)	Neck	Grazing or non-grazing (walking, standing, ruminating, drinking)	81 s	88.8%	45	Cattle	[18]
ACC, GYR	16–20 Hz	Ears, Neck	Walking, standing, lying, grazing	3–7 s	80–95%	6–23	Sheep	[20,21,22]
PRSs, IMU	30 Hz (PRSs), 200 Hz (IMU)	Claws of hind limbs	Gait analysis	\	\	10	Cattle	[44]
IMU, GPS	20 Hz (IMU), 1 Hz (GPS)	Chin, Neck, Hind legs	Feeding, ruminating, walking, standing, lying down	5 s	98.9–99.9%	22	Sheep	[15]

ACC = accelerometer, UR = ultrasonic transducer, GYR = gyroscope, PRSs = pressure sensors, GPS = global positioning system, IMU = inertial measurement unit.

IMUs play a crucial role in livestock behavior monitoring, with accelerometers being the most commonly adopted in research and commercial use. These sensors operate based on the principle that animal motion generates voltage signals proportional to changes in acceleration, reflecting the animal’s three-dimensional movement [45,46]. These sensors can measure real-time changes in animal movement along three axes, enabling the precise detection of static postures (standing, lying) through vertical acceleration patterns and dynamic movements (walking, running, jumping) through integrated acceleration profiles. Angular velocity measurements further enhance gait analysis and posture transitions detection, such as turning and tilting. By analyzing data on acceleration and angular velocity, researchers can effectively identify both the static behaviors (e.g., standing still, lying down) and dynamic behaviors (e.g., walking, running) of livestock.

Acoustic sensing infers behavior and emotional states by measuring characteristics such as frequency, amplitude, and duration of animal sounds, including vocalizations, breathing, and hoof sounds. This approach is particularly valuable for detecting stress indicators (e.g., increased vocalization frequency), feeding sounds (biting/chewing), and respiratory patterns. For instance, acoustic sensors can capture the sounds of livestock chewing and grazing, extracting features such as waveform, intensity, peak, and duration. When combined with algorithms, these features can accurately monitor and classify mandibular activity events in livestock [47], providing non-invasive insights into feeding efficiency and distress signals.

Pressure sensing was mainly used to monitor the jaw movement of livestock, and specific to recognize feeding and chewing behaviors of ruminants [42]. The pressure sensor is typically composed of sensitive components (such as resistive strain gauges, piezoelectric elements, etc.). When pressure is generated by animals (e.g., chewing), the sensor generates a corresponding electrical signal, which is proportional to the applied pressure. By transmitting these signals to a data-processing unit, real-time recording and analysis of pressure data can be conducted. These systems excel at quantifying rumination cycles and identifying abnormal chewing patterns indicative of health issues. Zehner et al. [41] assessed the performance of a noseband pressure sensor system, RumiWatch, on measuring ruminating and eating behavior. Showing a consolidated classification of sensor data, the correlations between sensor data and direct behavioral observations were 0.91–0.96 for rumination time, and 0.86–0.96 for eating time. The results showed that this system can provide farmers with sufficiently accurate data on feeding and rumination times, which can be used to support herd management decisions, particularly in early illness detection and nutritional management.

Positional sensing primarily tracks animal locations using GPS, Wi-Fi, or Bluetooth technologies, thereby inferring movement patterns and activity ranges. Currently, BDS/GPS technology is increasingly used in field monitoring [48,49,50]. By analyzing the movement paths of livestock on pastures, researchers can assess activity levels, resting areas, and social behaviors. This technology enables the mapping of grazing distribution, identification of preferential resting zones, and detection of estrus-related movement patterns. This information aids in understanding the foraging, social, and resting habits of grazing livestock (mainly cattle and sheep), supporting pasture rotation decisions and heat detection programs.

With technological advancements and the increasing demand for comprehensive management, these sensors enable four key applications: precision feeding management through rumination monitoring; estrus detection via activity and movement pattern changes; early disease identification such as lameness through gait analysis; and pasture utilization optimization. Single-type sensors are increasingly insufficient for complex monitoring needs. Different types of sensors each have their advantages. With technological advancements, researchers have begun to explore the integration of multiple sensors. Combining the motion information from accelerometers, vocalization patterns, emotional signals from acoustic sensors, and spatial movement data from positional sensors enables more accurate identification of various animal behaviors, such as feeding, resting, estrus, and social activities [51,52,53]. For example, while GPS technology can provide the location of livestock, it may be challenging to distinguish specific behavioral patterns, such as distinguishing between foraging, resting, and social interactions. Therefore, combining data from other sensors or observations is necessary for a more accurate interpretation of animal behavior. Brennan et al. [18] combined GPS trackers with three-axis accelerometers to monitor seasonal grazing behaviors in cattle, demonstrating how sensor fusion improves grazing efficiency assessment. Meanwhile, the integration of multiple sensors also presents challenges, particularly the conflict between low data acquisition frequency and long battery life. For wearable devices, there is normally limited resources for computing and power supply. It is essential to balance the need for prolonged device operation with achieving high precision in detection, especially in commercial farming operations where continuous monitoring is essential.

#### 3.1.2. Sampling Frequency

The sampling frequency of different types of sensors significantly affects the accuracy of animal behavior monitoring and energy consumption. An appropriate sampling frequency can reduce energy consumption while maintaining high accuracy [20,26,54,55,56].

For accelerometers, data sampling frequencies commonly range from 1 Hz to 50 Hz. Research by Walton et al. [20] indicated that the high sampling frequencies, such as 32 Hz, provide more detailed information on behavioral changes and are suitable for capturing complex behavioral features. However, this comes at the cost of increased energy consumption and data processing requirements. At a sampling frequency of 32 Hz with a 7 s time window, behavior classification in sheep achieved 95% accuracy and an F-score of 91–97%. In comparison, a sampling frequency of 16 Hz resulted in a slight decrease in accuracy to 91–93% and an F-score ranging from 88–95%, while the energy consumption was approximately 10 µA h for 16 Hz compared to 17 µA h for 32 Hz, demonstrating a good energy efficiency ratio.

Different behaviors may require different sampling frequencies for optimal classification results. For instance, Fogarty et al. [54] used a 12.5 Hz sampling frequency with a 10 s time window for classifying sheep behavior. They found lower classification accuracy for certain behaviors, such as lying and standing, but higher accuracy for walking behavior. When choosing the proper sampling frequency of sensors, the dynamics and complexity of the target behaviors should be considered. For dynamic behaviors such as walking and running, a higher sampling frequency may be necessary to capture rapidly changing features, whereas a lower frequency might suffice for static behaviors such as standing and lying down.

For acoustic and positional sensors, the existing literature suggests that acoustic sensors typically operate within a frequency range of 16 kHz to 44.1 kHz [57,58,59,60]. Due to the high sampling frequency, devices incorporating acoustic sensors require careful consideration of battery life. Positional sensors, which are often used in conjunction with accelerometers, do not have specific sampling frequency requirements and are generally set to meet the needs of battery life and tracking. Limited literature can be found on the sampling frequency of pressure sensors for behavior monitoring. According to unpublished data from the authors, a sampling frequency of 1 Hz is good enough to recognize the chewing behavior of cattle, but the sampling frequency needs to rise to 5 Hz to obtain satisfactory accuracy for calculating the chewing bouts.

#### 3.1.3. Sensor Deployment Position

A proper sensor deployment position significantly affects the effectiveness of behavior monitoring. Differing for varied monitoring purposes, the adopted deployment positions of wearable devices include the animal’s ears, forehead, jaw, neck, back, legs, and root of the tail [17,32,54,61,62]. Among these, the neck, legs, and jaw are the most commonly used in behavior monitoring.

Attaching accelerometers to the neck is a common practice to measure the activities of ruminants. Research by Guo et al. [62] and Cao et al. [63] showed that accelerometers placed on the neck of sheep generally provide superior identification efficiency compared to the position near the front legs or on the back, and the recognition accuracy for grazing behavior exceeding 97%. However, while the sensor is positioned on the neck, it is easy to shift under free-range conditions [64]. It should be assessed whether this would or would not affect the precise recognition of behavior in practical use. Additionally, the ears may be an alternative position. The results from Barwick et al. [32] indicate that accelerometers attached to the ears can differentiate lameness from grazing, standing, and normal walking. Integration with ear tags may be a potential use for ear-mounted sensors to monitor behaviors. It should be noted that it is easy to generate inertial noise, which would address potential data quality issues because there is no fixed point on the ear and the sheep moves fast [10]. Furthermore, deploying three-axis accelerometers or pressure sensors on the jaw provides the benefit of capturing detailed movements of mouth, enabling monitoring of fine feeding behaviors such as biting and chewing [61,65].

Acoustic sensors are typically mounted on the neck or forehead. For example, Shorten and Hunter [38] used a WS-853 digital recorder attached to the neck of dairy cows, achieving an overall accuracy of 99.5% in recognizing three different cow vocalization classes, which were open mouth, closed mouth, and mixed mouth (closed mouth followed by open mouth). Position sensors generally do not have specific placement requirements, as long as they do not interfere with normal animal activities and data collection. Currently, most positional sensors are integrated with other sensors, with the primary concerns on the placement of the other sensors.

Sensors mounted on the legs provide higher accuracy for posture and gait-related behavior recognition. When deploying three-axis accelerometers, the legs are the most stable location due to their rigid support. However, from an application perspective, the legs are not the optimal position [17,54]. This is because the leg-mounted sensors are only advantageous for recognizing lameness. Its recognition accuracies lowered for other behavior classifications compared to ear-mounted sensors and were not conducive to multifunctional integration applications.

### 3.2. Algorithms for Behavioral Recognition

**Figure 3:**
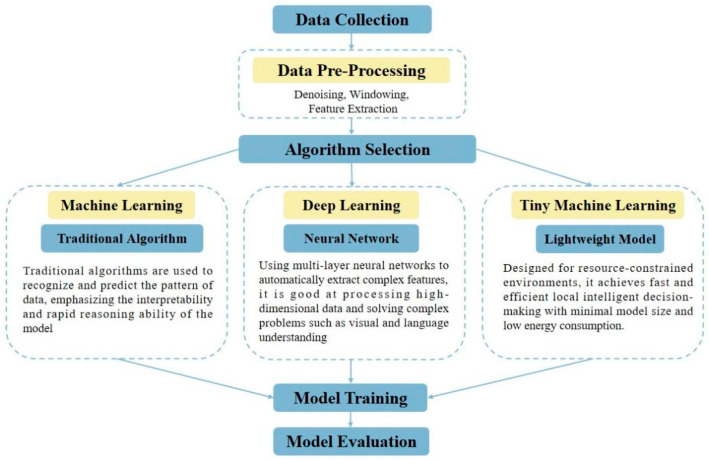
Overall framework for behavior recognition.

Models are essential for data processing and behavior recognition. In general, models for behavior monitoring based on wearable sensors follow a comprehensive framework, which includes several key stages: data pre-processing, algorithm selection for behavior classification, as well as model tuning and evaluation (as shown in Figure 3). Since the early stage of research to the present, machine learning has emerged as the predominant approach in this field [3,8,66]. This is primarily due to its consistent performance and relatively modest requirements for data volume. Traditional machine learning algorithms, such as Support Vector Machines (SVMs) and Random Forests, have been widely used for behavior classification. These algorithms are effective in recognizing and predicting patterns in the data, and they emphasize the interpretability of the model, which is crucial for understanding the reasoning behind the predictions. This has established machine learning as the mainstream methodology in the field [67].

As a technical improvement, deep learning has progressively emerged as a focal point of research, especially in image analysis. Deep learning algorithms, such as Convolutional Neural Networks (CNNs) and Recurrent Neural Networks (RNNs), leverage their potent ability to learn features from data automatically. They have shown superior performance in the analysis of intricate data sets, where traditional machine learning methods may struggle due to the complexity and high dimensionality of the data. Hence, algorithms for behavior recognition have undergone significant progress. Initially, traditional machine learning approaches were predominant. However, with the advancement of technology, deep learning methodologies have gained burgeoning ascendancy. In many cases, a combination of both approaches is employed to leverage the strengths of each. For example, traditional machine learning can be used for feature engineering, while deep learning can be utilized for complex pattern recognition.

In the data pre-processing stage, raw sensor data is cleaned and transformed to remove noise and irrelevant information. This may involve operations such as denoising, windowing, and feature extraction. Algorithm selection depends on the specific requirements of the behavior recognition task. For instance, if the data is in the form of images, deep learning algorithms like CNNs are more suitable. On the other hand, if the data is structured and the relationships between features are well-understood, traditional machine learning algorithms may be preferred.

Model tuning and evaluation are critical steps to ensure the performance of the models. This involves optimizing hyperparameters, such as learning rates and regularization parameters, and evaluating the models using appropriate metrics, such as accuracy, precision, recall, and F1-score. The evaluation results help in selecting the best-performing model for real-world applications. The evolution from traditional machine learning to deep learning, and the combination of both, reflects the continuous advancement in the field of behavior monitoring using on-animal sensors. This progress has enabled more accurate and efficient recognition of animal behaviors, which is essential for various applications in animal science and husbandry.

In addition to deep learning and traditional machine learning, a relatively new approach, Tiny Machine Learning (Tiny ML), is emerging in many fields with its unique advantages, especially in the application of edge devices like wearable sensors [68,69,70]. The deployment of models device-side through Tiny ML techniques significantly diminishes power consumption, compacts model dimensions, and ensures swift response times [71]. Although the field of Tiny ML remains in its nascent phase, it paves the way for novel applications in the realm of real-time monitoring and edge intelligent computing due to its advantages in conditions with limited resources. This suggests a promising trajectory for future integration within the domain of animal behavior recognition. This section introduced and compared the algorithms or methods used in the literature for data preprocessing and classification in behavior monitoring and discussed Tiny ML as an alternative way for behavior classification.

#### 3.2.1. Data Preprocessing

Data preprocessing is essential before implementing behavior recognition algorithms, as it augments the algorithms’ performance by refining data quality, standardizing data formats, and diminishing dimensionality. Furthermore, the processes of data cleaning and denoising are instrumental in bolstering the generalization capabilities of the algorithms. Concurrently, feature extraction plays a pivotal role in directing the algorithms’ attention to the most salient information pertinent to behavior recognition.

##### Denoising

Data collected by sensors in open environments invariably contains a certain degree of noise [72]. Therefore, the filtration and denoising of sensor data constitute an essential preliminary step in the construction of behavior recognition models [73]. The application of denoising techniques is necessary for refining the integrity of signal data within the context of behavior recognition algorithms. It serves to attenuate the computational burden by eliminating non-essential processing demands, thereby enhancing algorithmic efficiency. Concurrently, it reinforces the model’s resilience to fluctuations in environmental conditions, a critical factor in preserving the model’s predictive accuracy. Furthermore, denoising standardizes the data, which is instrumental in curbing the potential for overfitting, a common impediment to the generalizability of machine learning models [74]. The application of denoising techniques on behavior recognition is predominantly concentrated within the realms of the time domain, frequency domain, and time–frequency domain, extending their utility to the processing of waveforms, spectral data, and acoustic spectrograms [72]. Denoising strategies encompass a spectrum of techniques, with common practices including low-pass, band-pass, and high-pass filtering to attenuate unwanted noise components. In [75], low-pass filters with cutoff frequencies of 5 Hz and 10 Hz were used, as well as a high-pass filter with a cutoff frequency of 0.3 Hz without filtering. Sophisticated methodologies, such as optimal Finite Impulse Response (FIR) filters, spectral decimation, Minimum Mean Square Error Short-Time Spectral Amplitude Estimator (MMSE-STSA), and wavelet-based denoising, are deployed to further refine the signal-to-noise ratio [76]. Additionally, Wiener filtering and Singular Spectrum Analysis (SSA) have demonstrated particular efficacy in the context of bioacoustic signal processing, showcasing their utility in enhancing the clarity and integrity of acoustic data [72]. The selection of a denoising method should be adapted to the specific animal species and the objectives of the study [72]. Riaboff et al. [75] demonstrated the importance of choosing the filtering approach in a study recognizing cattle behavior. They found that high-pass filtering of acceleration signals significantly degraded classification performance, resulting in a 5% decrease in accuracy (from 92% to 87%) and a 6% decrease in the F-score (from 93% to 87%). The data denoising method used by Dineva et al. in the signal processing step is median filtering. The original signal is processed to reduce background noise, resulting in a smoother and clearer signal, which lays the foundation for subsequent feature extraction and behavioral pattern analysis. In addition, the literature also mentions the use of a low-pass filter (0.3 Hz) to process the acceleration signal, but the main purpose of this step is to separate the body movement component from the gravity component in the signal, rather than directly denoising [77]. Similarly, in the study by Arablouei et al., median filtering was also used to enhance robustness against outliers and noise [78].

##### Data Augmentation

In the research on animal behavior recognition based on wearable sensors, obtaining large-scale, high-quality, and balanced labeled datasets usually poses significant challenges. Data limitations (small data volume, imbalance, insufficient sample diversity, and missing values) directly affect the robustness and generalization ability of model training [79,80].

Current research commonly employs two primary techniques to address class imbalance, resampling, and cost-sensitive learning. Resampling directly adjusts the dataset by oversampling the minority class or undersampling the majority class (e.g., through random deletion of samples) to balance class distribution [81]. Cost-sensitive learning operates during model training by assigning different misclassification costs to different classes, typically imposing a higher penalty for misclassifying minority class instances compared to majority class instances. This can be achieved by adjusting the decision threshold or, more commonly, through class weighting techniques. The fundamental class weighting strategy introduces a weight coefficient, inversely proportional to class frequency, into the model’s loss function. This results in a higher loss for misclassifying rare minority class samples, thereby directing greater model attention towards minority classes [82]. This approach has been applied in numerous studies [83,84].

Generative Adversarial Networks (GANs) offer an alternative approach to address class imbalance by synthesizing minority-class samples. These synthetic instances are combined with the original data to construct balanced datasets, leveraging GANs’ capacity to learn latent data distributions and generate realistic samples [85]. This methodology enhances classifier performance on imbalanced datasets. However, in current animal behavior recognition based on wearable sensors, research on using generative models to handle missing data or imbalanced data is not yet common. This aspect can be considered for inclusion in future studies.

##### Windowing

Raw sensor data, which constitutes a continuous stream of information, can be effectively managed by partitioning it into manageable segments. This segmentation is crucial for capturing the nuances of local dynamics, simplifying computational demands, and responding to the fluidity of behavioral changes [86]. The strategy of employing static time windows, which are either mutually exclusive or exhibit overlap, presents distinct advantages. Non-overlapping windows provide discrete snapshots of behavior, while overlapping ones enhance the likelihood of capturing transitional phases [87]. It can be observed that window size typically ranges from 3 to 10 s and is selected by the particular requirements of the specific recognition task and the sampling frequency of sensors [46,88,89]. Shorter windows may be suitable for quickly identifying short-lived activities, while longer windows may be used to analyze more persistent or complex behavior patterns.

In the study by Nóbrega et al. [19], a 0.5 s window was employed to segment data sampled at 50 Hz and effectively captured the nuances of movement and rapid acceleration. It achieved a commendable accuracy of 91.78% in discerning behaviors such as infracting, moving, running, and standing of sheep using a decision tree algorithm. In contrast, Fogarty et al. [90] compared window sizes of 3, 10, and 30 s for data sampled at 12.5 Hz. Their findings suggest that larger windows are conducive to identifying behaviors that evolve over time, such as the shift from inactivity to activity. Employing a 30 s window, it attained an accuracy of 98.1% using the CART algorithm in recognizing general activities (active and inactive). This suggests that a longer time window helps to more accurately identify behavioral patterns that may take some time to unfold, such as transitions from a stationary to an active state. In contrast, there was an accuracy of 90.6% in discerning body postures using the Linear Discriminant Analysis (LDA) algorithm at a 30 s window [90]. These results indicate the importance of selecting an appropriate time window as a key determinant in the accuracy of behavior recognition.

##### Feature Extraction

Feature extraction is a vital technique used to enhance model performance. It refines the data representation by extracting and constructing new features from raw data to optimize the model’s capacity to discern underlying patterns and relationships [91]. In animal behavior recognition, this process is particularly important because it allows the model to understand and interpret complex patterns in the data in greater depth. Feature extraction improves the predictive power of models by reducing data dimensionality, capturing dynamic behaviors in time series, and enhancing the robustness of models to noise and outliers [92]. In addition, the flexibility of feature extraction allows researchers to explore the best representation of the data by trying different combinations of features, thus providing a solid foundation for building efficient and accurate classification models [93]. When performing feature engineering, it is usually approached from the time domain and the frequency domain to ensure that the intrinsic properties and dynamic behaviors of the data can be comprehensively captured.

Time-domain features can effectively reflect the dynamic fluctuations and evolving attributes of signals such as trends and periodic patterns. In time-domain analysis, feature extraction is concentrated on extracting information directly from time-series data, including but not limited to statistics (such as mean, variance, skewness, kurtosis), time-delay correlations (such autocorrelation coefficient), and other relevant features that can provide deeper insights into the data’s temporal dynamics [94]. The time-domain features usually adopted in animal behavior recognition can be found in Kleanthous et al. [35]. After feature extraction, it commonly follows feature dimensionality reduction or feature selection to remove irrelevant or redundant features and thereby improve the performance or interpretability of the model. Dimensionality reduction can be realized through methods such as principal component analysis (PCA) or LDA, and feature selection mainly includes filter, wrapper, embedded, and hybrid methods [35,92]. In the study of Guo et al. [62], 55 features were computed, including time-domain features such as maximum, minimum, mean, standard deviation, peak-to-peak, average frequency, energy, entropy, skewness, and kurtosis, and probabilistic principal component analysis (PPCA) was used for dimensionality reduction. The identifying accuracy of grazing behaviors exceeded 95% to reduce the feature space even when the 55 features were reduced to two principal components.

Conversely, frequency domain analysis centers on the transformation of time-domain signals into their frequency-domain counterparts. This process entails the application of various techniques to dissect the frequency components of the signals, thereby revealing insights into their cyclical behaviors and underlying structures. Methodologies usually adopted for the transformation include the Fourier Transform (FT), the Short-Time Fourier Transform (STFT), and the Wavelet Transform (WT) [95]. FT decomposes signals into their constituent frequencies. STFT is useful for non-stationary signals to analyze frequency content over time. WT offers time-frequency resolution and is adept at handling signals with varying frequencies over time. Frequency domain features help to reveal the distributional traits of data across various frequencies, which is invaluable for accurately locating and examining periodic patterns, identifying harmonic components, and characterizing noise elements within the data [96]. For example, Chen et al. [42] extracted the frequency-domain feature based on the FFT to recognize the feeding behaviors of cattle using noseband pressure. An accuracy of 96.6% is achieved using the XGB model.

#### 3.2.2. Behavior Classification

This article summaries and analyzes different algorithms on behavior classification according to their fundamental principles, such as tree-based models, distance-based models, Kernel and Linear models, Neural Network-based models, and assembled models.

##### Machine Learning Algorithm

Machine learning demonstrates significant effectiveness in extracting dynamic behavioral features while maintaining relatively high interpretability at low computational costs [97]. As evidenced in the figure in Table 4, various supervised algorithms including LDA, QDA, RF, CART, AdaBoost, and XGB remain predominant in animal behavior classification [35], though their performance reveals important patterns worthy of deeper consideration. Different machine learning algorithms indeed serve distinct purposes, though their practical implementation reveals noteworthy tradeoffs. While K-means effectively distinguishes static versus dynamic behaviors [63], and LDA/QDA offer valuable dimensionality reduction [54,98], the utility of distance-based models exhibited the most pronounced reduction for multi-behavior discrimination [17,98]. Tree-based models (RF, DT, XGB) demonstrate particular strength in balancing accuracy and generalization [57,99,100], exemplified by the 2.96% drop when expanding from four to five behaviors in RF implementations [66,101].

**Figure 4:**
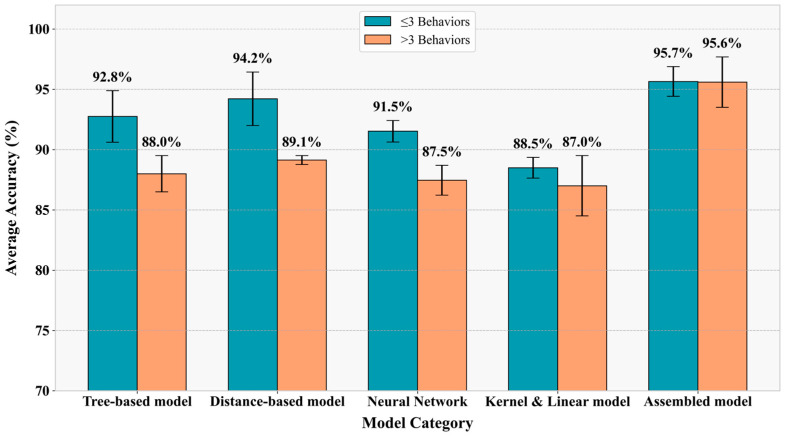
Comparison of the accuracy of different models when the recognized behaviors under of over three different types.

The quantitative evidence in Figure 4 consistently indicates an inverse relationship between the number of behaviors classified and achievable accuracy—a critical constraint not always addressed in methodology discussions. The observed accuracy degrades with increasing behavior categories. For instance, while simple models like FMM+DT achieve exceptional accuracy (99%) for basic postures in small cohorts, RF shows substantially reduced performance (76–94%) when classifying eight complex behaviors despite moderate data volume. This pattern extends across methodologies: traditional machine learning algorithms achieved only moderate accuracy (79–93%) on behavior classification despite exceptionally large datasets, suggesting that data quantity alone cannot overcome fundamental algorithmic limitations for complex recognition tasks. This limitation has naturally driven exploration of both algorithm cascading and deep learning solutions, though each approach carries distinct computational and practical implications. Consequently, the field has progressively shifted toward ensemble algorithms and neural networks.

##### Algorithm Assembling and Deep Learning

Table 4 collectively provides a methodologically stratified comparison of computational approaches in livestock behavior recognition, tracing an evolutionary trajectory from conventional machine learning to deep neural networks. Crucially, assembled machine learning models fill a critical gap often overlooked in prior reviews by documenting hybrid ensemble methods that bridge traditional and deep learning paradigms.

Algorithm assembling can make full use of the advantages of various algorithms, improving the overall performance over a single algorithm. Some common ensembled algorithms are also shown in Table 4. The fusion of traditional algorithms like FA with SVM reaches 98.02% accuracy while processing substantial datasets of 4.2 million samples, indicating remarkable scalability for agricultural applications [99]. The Hidden Markov Model (HMM) deals with time-varying characteristics in time-series data, adding temporal continuity to separate time windows [115]. It was applied to correct the recognized behaviors with the state transition probability matrix after behavior classification using XGB or RF [116]. Results from Ding et al. demonstrate that the accuracy would be greatly enhanced (about 5–10%) after applying the HMM-Viterbi algorithm integrated with XGB to identify the chewing and ingesting behaviors from jaw movement through a triaxial accelerometer [111]. These ensemble strategies effectively balance computational demands with real-world applicability, particularly evident in how GA-SVM maintains 95–98% accuracy across eight distinct ewe behaviors with under 30,000 samples [26]. This efficiency profile positions such methods as operationally viable for farm environments where computational resources are often constrained. Other examples are the K-Means clustering algorithm for behavioral categorization and the SVM algorithm to identify different feeding stages to improve the classification accuracy of fine feeding movement such chewing [3,9]. Besides algorithm assembling of traditional machine learning approaches, deep learning is capable of automatically learning and extracting complex features from data to improve the accuracy and robustness of behavioral classification.

In theory, deep learning is a subset of machine learning that automatically extracts complex features of data by utilizing multi-layer neural networks. Developed in part to improve computational efficiency and handle more complex data patterns, deep learning demonstrates its advantages in understanding complex behavioral patterns by increasing the depth and width of the model to learn more abstract feature representations [97,117]. For example, Convolutional Neural Network (CNN) and Long Short-Term Memory Network (LSTM) have been used to detect cattle behavior by collecting behavioral data through wearing IMU sensors, extracting and fusing features from different parts of the body, and realizing high-precision behavioral analysis and individual identification [67]. Recurrent Neural Network (RNN) and its variant LSTM are suitable for recognizing long-term dependencies in sequential data such as consecutive actions or behavioral patterns such as walking, running, etc., and achieved an accuracy of 92.6% [67]. While architectures like GAN-TCN achieve impressive 97.15% accuracy in recognizing complex goat locomotion patterns, they require substantially larger datasets (211,000 samples) compared to ensemble approaches [100]. The consistency challenge emerges clearly in grazing behavior recognition, where CNN-BP networks exhibit significant accuracy variance (83–94%) despite substantial data inputs [55]. Furthermore, specialized architectures like Conv1D-Conv2D-LSTM achieve 93% accuracy for acoustic-based chewing detection but remain confined to laboratory validation with only three animals, highlighting scalability limitations [118]. Although deep learning shows great potential for automatic feature extraction, it requires a large amount of computational resources and data to train the model [97]. Therefore, it normally served as cloud machine learning, and the success of deep learning models often comes at the cost of high computation, which is not feasible for using an edge device. The choice between traditional machine learning or deep learning approaches in behavioral recognition research and applications usually depends on the specific task requirements, the nature of the data, and the available computational resources [97].

#### 3.2.3. Promising Use of Tiny Machine Learning

**Figure 5:**
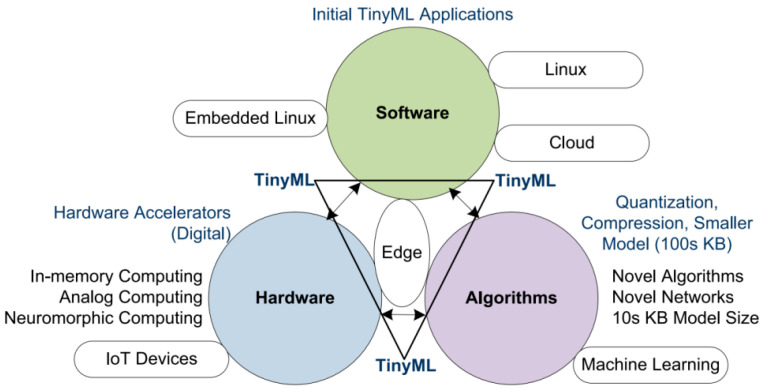
Composition of Tiny ML (Ray, 2022 [71]).

Tiny ML is a new frontier of machine learning and represents an adaptive approach tailored for environments with limited computational resources [119]. It targets predominately to battery-operated embedded edge devices and has many practical applications including personalized healthcare, wearable or IoT devices, smart home, ecology, and agriculture [120]. As shown in Figure 5, Tiny ML consists of the three key elements of software, hardware, and algorithms, which can be accommodated in Linux, embedded Linux, and cloud-based software [71]. Tiny ML requires specific libraries and software platforms to support its use. The existing software tool includes uTensor [121], Edge Impulse [122], NanoEdge AI Studio [123], STM32Cube.AI [124], etc. By implementing the Tiny ML framework on an Arduino Uno board, it can be demonstrated that complex models like neural networks, SVM, decision trees (DTs), and RF are still feasible on resource-constrained devices with accuracy close to that of standard models [125].

Recent pilot studies suggest Tiny ML’s emerging potential for animal behavior monitoring, demonstrating notable improvements in embedded system efficiency relative to conventional approaches. Swine vocalization classification systems utilizing convolutional neural networks on Cortex-M4 processors have attained >90% accuracy in detecting agonistic and social behaviors, enabling real-time welfare interventions [126]. Similarly, multi-modal architectures fusing accelerometer and vision data for cattle behavior recognition exhibit 270× model compression and sub-80 ms inference latency when deployed on ESP32 microcontrollers [127]. These advances are facilitated through meticulous hardware–algorithm co-design, incorporating architectural optimization frameworks like Edge Impulse and deep compression techniques (pruning/quantization) that maintain performance parity while eliminating cloud dependency—reducing analysis latency from minutes to milliseconds. Beyond direct behavior monitoring, Tiny ML enhances broader applications including aquaculture anomaly detection via LoRaWAN-based water quality monitoring and egg quality assessment, achieving 95.8% accuracy on Arduino Nano platforms and environmental prediction [69,70]. Consequently, Tiny ML has proven transformative for efficient on-site animal monitoring, with its edge-computing paradigm addressing critical constraints in power budgets and data transmission while sustaining high-precision analytics.

## 4. Application with Behavioral Monitoring

### 4.1. Feed Intake Estimation

Feed intake is an important indicator that helps optimize daily management and improve the production efficiency of livestock [128]. Contact sensors, such as acceleration sensors installed on the neck or jaw of cattle, can estimate feeding information such as feed intake, feeding rate, and feeding duration, by monitoring the animal’s feeding behavior in real time [2,111]. Compared to the trough scale, on-facility modifications are required using this approach, and it can be integrated for multi-function use (e.g., estrus alarm, early detection of health problems in animals) using the continuous data stream provided by one set of sensors. In addition, analyzing the amount of feed intake and behavioral patterns of animals with wearable sensors can subsequently assess the grazing capacity of meadows for the proper use of pasture and improve the production efficiency of grassland [10].

Existing feed-intake estimation models were mostly achieved by mathematical model fitting, i.e., static models. From early studies, the feed intake of grazing livestock is typically estimated through identified grazing behavior by the regression of behavior indicators such as chewing bouts, bite rate, feeding duration, ruminating duration, etc. [64,129,130] For example, Shangru et al. detected the feeding time and rumination time of 10 cows with wearable collars to estimate their feed intake using different algorithms including linear regression (LRM), ANN, SVM, and KNN [4]. Indicating a nonlinear relationship between feed intake and feeding time and ruminating time, LRM showed an R2 of 0.73, while the other models showed the R2 greater than or equal to 0.82. For another example, Davison et al. estimated the feed intake of eighty cows using four modeling approaches, including the proportion of the total feeding time of individual animals (GRP), multiple linear regression (REG), RF, and SVM [131]. The RMSE of dry matter intake (DMI) estimation of the REG, RF, and SVM ranged from 1.15 to 1.61 kg, whereas a higher error was observed using GRP due to the fact that it did not capture the individual feeding patterns.

Estimating the feed intake of livestock using behavior quantification indicators is often limited by the segmentation and accuracy of dynamic quantification of feeding behaviors, especially when dealing with complex feeding behaviors such as mixed movements of feeding, chewing, and swallowing [3]. Hence, behavior monitoring needs to be further promoted to synchronization recognition of multi-category behaviors (fine behaviors like chewing, biting, and regular behaviors like feeding, ruminating) and the quantification of behavior indicators. Furthermore, in combination with positional sensing, especially for grazing animals, the movement patterns of animals in an enclosure or feeding area can be analyzed and fused with data on feeding behavior to improve the accuracy of feed intake estimation. For example, the duration and intensity of feeding behavior can be inferred by analyzing the duration of an animal’s residence time and activity patterns near the feed trough [3].

### 4.2. Estrus and Parturition Alarming

Behavioral monitoring is a useful tool in the reproduction management of livestock for early identification of estrus and parturition. Contact sensors can provide a rich data stream over relatively long periods of time to capture their behavioral movements and sound, which in turn can be used to assess the physiological status of animals [132]. For example, Talukder et al. measured the activity and rumination of lactating cows by wearing accelerometers and microphones to identify factors associated with estrous events using a multivariate logistic generalized linear mixed model [5]. Assessing the overall utility by plotting of the ROC curve and calculating the area under the curve (AUC), the results showed that AUC was 0.82 for activity level, 0.54 for rumination level, and 0.75 for the combination of activity and rumination. This indicates that the activity level generated a more accurate estrus alarming. In the study of Silper et al. [6], the activities of cows were detected by two contact sensors (Heatime and IceTag) to detect signs of estrus at a high activity level. At a baseline of 87 ± 28 steps per hour, IceTag recorded 371 ± 91 steps per hour during estrus. Compared to a 360 ± 170% increase in activity relative to walking activity, it resulted in a positive estrus alarming of 98.7%, while the Heatime recorded an estrus intensity of 77.3 ± 19.5 peak exponential values, resulting in a positive estrus alarming of 84.7%.

Acoustic sensing plays an important role in livestock estrus monitoring, although for some reason it has not yet been commercially used in production. Research showed that the characteristics of sound such as intensity and frequency changes in estrus for cows and ewes [133,134]. Combined with machine learning algorithms such as K-Means clustering and SVM, automated estrus status recognition can be achieved. One of the reasons why acoustic sensing has not been widely used in production is due to the relatively large requirement of computation resources of acoustic analysis and the difficulty of implementation in edge devices. The further application of Tiny ML may be a solution in these resource-constrained scenarios [119].

Aside from estrus alarming, behavior monitoring can also be used for parturition warning [135]. This would be important for grazing animals, as it could help to reduce the mortality of newborn animals. For example, Smith et al. tried to identify the parturition of pregnant ewes based on the changes of their activity levels by either Maximum Mean Discrepancy (MMD) or the Earth Mover’s Distance (EMD) [136]. The results from 76 pregnant ewes showed that these algorithms can estimate the birth of lambs, with 84% of parturition events falling within 12 h of the actual birth time, while their study also showed that models need to be developed over sufficiently long periods (2 h ≤ time period ≤ 6 h) to capture the feature and enhance the model performance. 

### 4.3. Assessing Animal Health and Welfare

The health status of animals is crucial in farm management, not only affecting productivity but also being an important consideration for animal welfare. Behavioral changes are good indicators of abnormalities caused by internal or external stimuli in animals. It helps to detect early signs of disease, and by identifying abnormal behaviors such as loss of appetite, reduced activity, or changes in social behavior, potential health problems can be detected early for timely intervention [7,9]. A study by Wang et al. indicated that pig behavior and vocalizations can be monitored in real time and translated into key health indicators, enabling the early detection and intervention of diseases, and improving productivity and animal welfare in pig farms [9]. In addition, combining multiple sources of data such as accelerometers, temperature sensors, and heart rate monitors allows for the construction of more comprehensive health status metrics that can be used to identify health abnormalities in animals in real time through anomaly detection algorithms [137]. In this context, contact sensors provide a useful tool for the assessment of animal health and welfare.

Lameness is a common disease in ruminants caused by hoof lesions, limb lesions, or defects in locomotor and results in loss of animal production. A study from Norring et al. showed that cows with more severe lameness had shorter feeding times per day (lameness scores of 2, 3, and 4, 104 ± 4 min, 101 ± 4 min, and 91 ± 4 min/day, respectively) and consequently reduced the milk yield of cows [138]. Hence, lameness identification helps reduce production loss, and this can be realized through behavior monitoring using contact sensors [8]. For example, Chapinal et al. investigated the effectiveness of acceleration of the legs and back of dairy cows to detect changes in gait or locomotion associated with lameness [139]. The results showed that the asymmetry of variance of acceleration within a pair of legs was correlated, and accelerometers attached to a leg would be a promising tool for lameness detection on farm.

Diseases for the digestive system, such as subclinical ketosis and subclinical acidosis, affect a large percentage of dairy cows and have a negative impact on their performance [140]. The results from Antanaitis et al. showed that cows with subclinical ketosis (SCK) or subclinical acidosis (SCA) would significantly decrease 17.47% of the rumination time, and 36.84% of the eating time, 38.10% of the eating chews, and lead to a 27.36% reduction in the overall activity levels compared to the healthy cows [141]. This indicates the promising application of behavior monitoring for the early detection and effective management of SCK and SCA on dairy cow health [142].

Besides these examples mentioned above, there may still be some other potential application scenarios for assessing animal health with behavior monitoring. However, health assessment with behavior monitoring still faces many challenges. First, data complexity and noise handling are key issues, especially when fusing multiple sensors and cross-analyzing data from multiple sources. Second, how to translate the monitoring results into practical health management strategies and how to maintain the consistency and accuracy of monitoring across environments and species also need to be further studied.

## 5. Challenges and Prospects

### 5.1. Current Challenges

Significant progress has been achieved in the monitoring and application of livestock behavior based on contact sensors, whereas numerous challenges remain to be addressed.

Firstly, the processing and interpretation of data remain complex and not feasible for edge computation in practical use. The algorithms are generally established with manual feature extraction and are based on fixed rules or training datasets, but often have limitations in the segmentation of dynamic behaviors and the adaptability to complex scenes [37,143]. In the scene of wearable devices, optimized or miniaturized algorithms should be established to adapt to the limited computing resources of wearable devices. Although the new method of Tiny ML technology can execute machine learning models on low-power devices, significantly improving the energy efficiency and response speed of the system [7,71]. It is still an urgent issue about how to achieve a balance between real-time preprocessing, feature extraction, and the model selection of data to ensure recognition accuracy and reduce computational complexity [7].

Secondly, while many researchers use three-axis acceleration sensors to identify livestock behavior, most of the models have not been systematically assessed and validated with embedded systems in practical application, including the effectiveness of behavior recognition and the power consumption or maximum endurance [144]. Existing behavior identification models rely on high-frequency data acquisition and large-scale data transmission. This would result in high power consumption and short endurance of wearable devices. Algorithms for precision behavior recognition at a low frequency of data acquisition are needed to reduce resource loss in data transmission and storage and maintain the identification accuracy.

Finally, the reliability and cost of wearable devices pose another challenge. The environmental condition and sensor placement effects on data quality is a critical understudied aspect of real-world deployment. The durability under different environmental conditions, along with their installation and maintenance costs of wearable devices, have hindered their widespread application. For example, sensors may affect the accuracy and consistency of data due to wear, tear, and self-cleaning of animals or harsh weather conditions. These interdependent factors create unstandardized error sources that currently prevent systematic quantification. Appropriate validation methods need to be established to ensure reliable data collection in real-world settings. In addition, the integration of sensors and the fusion of data, especially in the case of multimodal data, require more advanced algorithms to reduce noise, improve the efficiency of feature selection, and ensure consistency across sensor data [8,145].

### 5.2. Future Research Prospects

Despite the above challenges, looking into the future, there is a considerable prospect for livestock behavior monitoring and application based on wearable sensors or devices. The integrated use of sensors will promote the multi-function application and improve the accuracy of behavior monitoring. For example, studies can explore more sensor fusion strategies, such as combining physiological parameters (such as heart rate and body temperature) with motion characteristics to achieve a more comprehensive health assessment [137].

In terms of model construction, the aforementioned literature mentioned above indicates that the classification accuracy of routine livestock behavior is relatively high. However, when applying features of daily behavior, the performance is not as satisfactory as expected with the increase in the number of test samples. Predicting individual characteristics of complex behaviors typically exhibits lower efficiency and weaker model robustness. Therefore, in future research, strategies such as enhancing data diversity, integrating multimodal data, applying data augmentation techniques, introducing robustness regularization, and implementing cross-domain transfer learning could be considered to enhance the model’s predictive efficiency and robustness for individual characteristics of complex behaviors, thereby advancing the model’s generalization capabilities and stability. In addition, optimized or compressed models suitable for edge computing of wearable devices are needed, as mentioned above.

Translating behavior monitoring results into practical management decisions is a hot topic in current research. Although changes in behavior patterns can indicate an animal’s health or productivity, translating this information into specific, actionable advice can be challenging for farmers. Interdisciplinary collaboration, such as the integration of biology, computer science, and engineering, is crucial for developing refined management strategies based on monitoring results. For example, the Five Domains Model proposed by Fogarty et al. [146] can be used as a framework for evaluating animal welfare, but further research and development are still needed to integrate sensor data with each domain of the model. It is emphasized that all studies referenced herein operate under institutional animal ethics protocols. Future advances must continue prioritizing miniaturization, energy efficiency, and non-invasive designs to further align technical innovation with animal welfare imperatives.

## Figures and Tables

**Table 4:** Models adopted normally in livestock behavior recognition.

Category	Model	Reference	
Tree-based model	RF, GBDT, DT, XGB	[20,102,103,104,105]	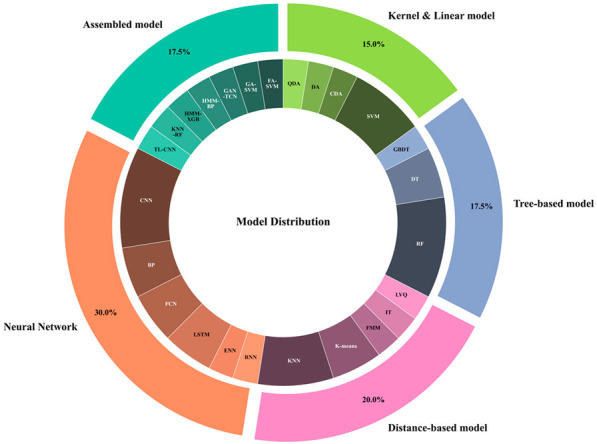
Distance-based model	K-means, KNN, FMM, LVQ, IT	[63,98,105,106,107]	
Kernel and Linear-based model	CDA, DAQ, DA, SVM	[17,61,65]	
Neural Network-based model	BP, CNN, RNN, FCN, ENN, LSTM	[55,108,109,110]	
Assembled machine learning model	KNN-RF, FA-SVM, GA-SVM, HMM, HMM-XGB/BP, GAN-TCN, CNN-TL	[26,39,57,99,100,111,112,113,114]	

IT = Interval Thresholding Classification, CDA = Classical Discriminant Analysis, DA = Discriminant Analysis, FMM = Finite Mixture Models, KNN = K-Nearest Neighbors, LVQ = Learning Vector Quantization, SVM = Support Vector Machine, DT = Decision Tree, RF = Random Forest, GBDT = Gradient-Boosting Decision Tree, HMM = Hidden Markov Model, FA = Firefly Algorithm, GA = Genetic Algorithm, BP = Backpropagation Neural Network, FCN = Fully Convolutional Network, CNN = Convolutional Neural Network, LSTM = Long Short-Term Memory, RNN = Recurrent Neural Network, TL = Transfer Learning, GAN = Generative Adversarial Network, TCN = Temporal Convolutional Network.
